# A comprehensive dataset on antimicrobial activity of indigenous *Oscillatoria* spp. against common bacteria causing diseases in fish and shellfish

**DOI:** 10.1016/j.dib.2023.109563

**Published:** 2023-09-12

**Authors:** Jannatul Nayeem, Sumit Kanti Dey, Proma Dey, Inkiad Ahmed Himel, Helena Khatoon

**Affiliations:** Department of Aquaculture, Chattogram Veterinary and Animal Sciences University, Chattogram 4225, Bangladesh

**Keywords:** Cyanobacteria, *Oscillatoria*, Pathogenic bacteria, Antimicrobial activity, Minimum inhibitory concentration, Activity index

## Abstract

The antibacterial activity of phenolic extracts was assessed using the disk diffusion technique on two marine and two freshwater species of *Oscillatoria*. Three Gram positive bacteria (*Staphylococcus* sp. and *Streptococcus* sp.) and nine Gram negative bacteria (*Escherichia coli, Vibrio* spp., *Salmonella* sp., *Shigella* sp., *Pseudomonas* spp., *Aeromonas* sp.) were isolated from diseased marine fish, shrimp and crab. Filamentous *Oscillatoria* sp. indicated higher antibiotic activity than planktonic one. Gram positive bacteria showed higher susceptibility to phenol compared to the gram-negative bacteria. Filamentous *Oscillatoria* sp. showed highest inhibition zone of 34.06 ± 0.08mm against *Staphylococcus* sp., while planktonic *Oscillatoria* sp. showed lower inhibition zone against *Pseudomonas* sp. about 17.11 ± 0.18mm. Minimum inhibitory concentration value was found to be 100 µg/ml for filamentous *Oscillatoria* sp., 150 µg/ml for planktonic *Oscillatoria* sp. These findings suggest that, *Oscillatoria* spp. contain potential antibacterial substances. It also paves the way for detailed analysis of *Oscillatoria* spp. bioactive compound for the creation of novel antibiotics and serve both the aquaculture and pharmaceutical industries.

Specifications Table

**Table:** 

Subject	Agricultural Science, Aquatic Science

Specific subject area	Antimicrobial activity of indigenous cyanobacteria against common bacteria causing diseases in fish and shellfish
Type of data	Graph and Table
How the data were acquired	Data on antimicrobial activity test were collected through disk diffusion method. Bacteria were isolated by selective agar media streaking, Gram's staining, biochemical and VITEK-2 test. MIC value was determined by ELISA test using spectrophotometer (630nm). The collected data were analyzed by using MS Excel and IBM SPSS (v. 26.0) software.
Data format	Raw and analyzed
Description of data collection	For Sensitivity test: Disk diffusion assay For isolated bacteria: Gram's staining, Biochemical test and VITEK-2 system For MIC value: Tube micro dilution technique
Data source location	Disease and Microbiology laboratory, Department of Aquaculture, Chattogram Veterinary and Animal Sciences University (CVASU), Khulshi-4225, Chattogram, Bangladesh
Data accessibility	Data are available with this article and also at https://data.mendeley.com/datasets/bc7v9hsv82/1

## 1 Value of the Data


 

• Findings of this study supported the possibility of treating pathogenic bacterial diseases in fish and shellfish by means of *Oscillatoria* sp. extracts.
• Cyanobacterial phenolic compounds exhibit the potentialities to exploit them as valuable resource for the development of novel antimicrobial agents which will help to boost up the production of aquaculture industry.
• These data might create the future prospectives for the further research on antifungal and anti-cancerous properties based on detailed bioactive compound analysis of *Oscillatoria* sp.

## 2 Data Description

Pathogenic bacteria were isolated from the diseased marine fish and shellfish (Shrimp, Crab) (Table 1) [1]. Each isolated bacterium was cultured in their respective selective agar medium. Table 1 represents the isolated bacteria with its source, growth media and type.

**Table 1:** Isolated pathogenic bacteria from marine diseased fish and Shellfish.

Sl. no	Bacteria name	Source	Growth observation	Gram Staining
1	*Escherichia coli*	Tilapia (*Oreochromis mossambicus*)	Pink colonies on MacConkey agar	Gram-negative rod
2	*Salmonella* sp.	Crab (*Scylla serrata*)	Opaque colonies on SS agar	Gram-negative rod
3	*Shigella* sp.	Tuna (*Euthynnus affinis*)	Black colonies on SS agar	Gram-negative rod
4	*Pseudomonas* sp. 1	Gangetic Hairfin Anchovy (*Setipinna phasa*)	Opaque colonies on Pseudomonas agar	Gram-negative rod
5	*Pseudomonas* sp. 2	Rita (*Rita rita*)	Greenish-blue colonies on Pseudomonas agar	Gram-negative rod
6	*Vibrio* sp. 1	Shrimp (*Penaeus monodon*)	Green colonies on the TCBS agar	Gram-negative curved rod
7	*Vibrio* sp. 2	Tuna (*Euthynnus affinis*)	Greenish colonies on TCBS agar	Gram-negative curved rod
8	*Vibrio* sp. 3	Shrimp (*Penaeus monodon*)	Yellow colonies on TCBS agar	Gram-negative curved rod
9	*Aeromonas hydrophila*	Poa (*Otolithoides pama*)	Green colonies on TCBS agar	Gram-negative rod
10	*Streptococcus* sp.	Tilapia (*Oreochromis mossambicus*)	Opaque colonies on Nutrient agar	Gram-positive cocci
11	*Staphylococcus* sp. 1	Poa (*Otolithoides pama*)	Yellow colonies on Mannitol Salt agar	Gram-positive clusters
12	*Staphylococcus* sp. 2	Crab (*Scylla serrata*)	Pink colonies on Mannitol Salt agar	Gram-positive clusters

All *Oscillatoria* spp. extracts showed higher zone of inhibition and the variations of their zone found statistically significant (p < 0.05). Comparatively, higher inhibition zone (mm) was obtained from *Oscillatoria* sp. 2 (24.59 ± 0.21 to 34.05 ± 0.13) and *Oscillatoria* sp. 4 (26.80 ± 0.21 to 33.94 ± 0.15) whereas lower inhibition zone (mm) was recorded from *Oscillatoria* sp. 3 (21.52 ± 0.19 to 30.20 ± 0.18) and *Oscillatoria* sp. 1 (20) (17.11 ± 0.15 to 26.27 ± 0.15). Significant variations of *Oscillatoria* strains are demonstrated in Table 2.

**Table 2:** Diameter of the inhibition zone (mm) exhibited by *Oscillatoria* species against isolated pathogenic bacteria. Values are expressed as mean of the six replicates with standard error (mean ±  SE) and values with the different letters within each series indicate significant differences (p < 0.05) among the species. Concentration of phenolic extracts of *Oscillatoria* spp. were 30µl/disc. Colistin (10µg) and Ceftriaxone (30 µg) were used for reference antibiotic for Gram's negative and Gram's positive bacteria respectively.

Bacteria	*Oscillatoria* sp. 1	*Oscillatoria* sp. 2	*Oscillatoria* sp. 3	*Oscillatoria* sp. 4	Antibiotic
*E. coli*	22.86 ± 0.18^cd^	30.47 ± 0.20^b^	26.07 ± 0.24^d^	27.69 ± 0.22^cde^	17.22 ± 0.16^bc^
*Salmonella* sp.	22.42 ± 0.19^d^	30.13 ± 0.11^b^	27.58 ± 0.11^c^	29.72 ± 0.20^b^	17.31 ± 0.11^b^
*Shigella* sp.	24.85 ± 0.12^b^	34.00 ± 0.17^a^	30.20 ± 0.18^a^	33.94 ± 0.15^a^	16.62 ± 0.14^cde^
*Pseudomonas* sp. 1	22.79 ± 0.23^cd^	28.75 ± 0.12^c^	27.16 ± 0.23^c^	27.78 ± 0.18^cd^	15.92 ± 0.19^f^
*Pseudomonas* sp. 2	17.11 ± 0.15^g^	33.96 ± 0.20^a^	26.05 ± 0.10^d^	27.87 ± 0.19^cd^	17.07 ± 0.13^bcd^
*Vibrio* sp. 1	25.10 ± 0.27^b^	33.26 ± 0.26^a^	25.53 ± 0.21^de^	28.59 ± 0.14^c^	17.49 ± 0.04^ab^
*Vibrio* sp. 2	20.58 ± 0.19^e^	30.12 ± 0.19^b^	22.57 ± 0.24^f^	30.06 ± 0.17^b^	16.52 ± 0.15^def^
*Vibrio* sp. 3	18.05 ± 0.14^f^	33.88 ± 0.12^a^	21.90 ± 0.21^fg^	26.80 ± 0.21^e^	16.38 ± 0.06^ef^
*Aeromonas hydrophila*	19.84 ± 0.14^e^	29.91 ± 0.16^b^	21.52 ± 0.19^g^	27.23 ± 0.17^de^	15.97 ± ± 0.11^ef^
*Streptococcus* sp.	26.27 ± 0.15^a^	24.59 ± 0.21^d^	24.76 ± 0.17^e^	27.76 ± 0.24^cd^	14.78 ± 0.18^g^
*Staphylococcus* sp. 1	20.60 ± 0.21^e^	33.57 ± 0.07^a^	25.98 ± 0.09^d^	29.53 ± 0.18^b^	16.03 ± 0.1^ef^
*Staphylococcus* sp. 2	23.44 ± 0.14^c^	34.05 ± 0.13^a^	28.83 ± 0.15^b^	33.19 ± 0.20^a^	18.08 ± 0.10^a^

Activity index of this study shows significant (p < 0.05) variations among all the *Oscillatoria* strains (Table 3). Significantly (p < 0.05) higher activity index (AI) was reported from Strain 2 M2 (25) (2.10 ± 0.03) against *Staphylococcus* sp. 1 and the lower AI value was obtained from Strain 1 M1 (20) (1.00 ± 0.01) against *Pseudomonas* sp. 2.

**Table 3:** Antimicrobial index (AI) (mean ±  SE) of *Oscillatoria* strains against isolated pathogenic bacteria. Values with the distinct letters in each series indicate significant distinctions (p < 0.05) among the *Oscillatoria* species.

Bacteria	*Oscillatoria* sp. 1	*Oscillatoria* sp. 2	*Oscillatoria* sp. 3	*Oscillatoria* sp. 4
*E. coli*	1.33 ± 0.02^c^	1.77 ± 0.02^f^	1.51 ± 0.03^de^	1.61 ± 0.02^f^
*Salmonella* sp.	1.30 ± 0.01^cd^	1.74 ± 0.01^fg^	1.59 ± 0.01^cd^	1.72 ± 0.02^de^
*Shigella* sp.	1.50 ± 0.01^b^	2.05 ± 0.02^ab^	1.82 ± 0.02^a^	2.04 ± 0.02^a^
*Pseudomonas* sp. 1	1.43 ± 0.02^b^	1.81 ± 0.02^ef^	1.71 ± 0.02^b^	1.75 ± 0.02^cd^
*Pseudomonas* sp. 2	1.00 ± 0.01^f^	1.99 ± 0.02^bc^	1.53 ± 0.02^de^	1.63 ± 0.02^ef^
*Vibrio* sp. 1	1.44 ± 0.02^b^	1.90 ± 0.02^cd^	1.46 ± 0.01^e^	1.63 ± 0.01^ef^
*Vibrio* sp. 2	1.25 ± 0.02^d^	1.82 ± 0.02^ef^	1.37 ± 0.02^f^	1.82 ± 0.02^bc^
*Vibrio* sp. 3	1.10 ± 0.01^e^	2.07 ± 0.01^ab^	1.34 ± 0.02^f^	1.64 ± 0.01^ef^
*Aeromonas hydrophila*	1.24 ± 0.01^d^	1.87 ± 0.01^de^	1.35 ± 0.01^f^	1.71 ± 0.02^def^
*Streptococcus* sp.	1.78 ± 0.02^a^	1.67 ± 0.03^g^	1.68 ± 0.03^bc^	1.88 ± 0.04^b^
*Staphylococcus* sp. 1	1.29 ± 0.02^cd^	2.10 ± 0.03^a^	1.62 ± 0.02^bc^	1.84 ± 0.03^b^
*Staphylococcus* sp. 2	1.30 ± 0.01^cd^	1.88 ± 0.01^de^	1.59 ± 0.01^cd^	1.84 ± 0.01^bc^

### 2.1 Minimum Inhibitory Concentration (MIC)

The Minimum Inhibitory Concentration (MIC) value of each filamentous *Oscillatoria* species (*Oscillatoria* sp. 2, *Oscillatoria* sp. 3, *Oscillatoria* sp. 4) was found to be 100 µg/ml against all the test bacteria. On the contrary, planktonic strain (*Oscillatoria* sp. 1) required MIC value of 100 µg/ml to be effective against all the test bacteria.

## 3 Materials and Method

### 3.1 *Culture of Oscillatoria* sp

#### 3.1.1 Collection and preparation of pure inoculums

Pure isolates of two freshwater and two marine water *Oscillatoria* sp. obtained from Live Feed Culture laboratory of Department of Aquaculture, Faculty of Fisheries, Chattogram Veterinary and Animal Sciences University, Chattogram, Bangladesh. Isolates were inoculated in the Bold's Basal Medium (BBM) and Conway medium using the formula described by Bischoff and Bold. (1963) [2] and Tompkins et al. (1995) [3] respectively. Hundred ml pure seed of *Oscillatoria* sp. was inoculated into 900 ml of culture medium for the stock culture maintenance. Then the cultures of *Oscillatoria* sp. 1 (planktonic; marine), *Oscillatoria* sp. 2 (highly filamentous; marine), *Oscillatoria* sp. 3 (moderate filamentous; freshwater), *Oscillatoria* sp. 4 (highly filamentous; freshwater) were incubated in controlled indoor condition at 24 °C temperature for 13 days, 10 days, 11 days, 9 days respectively. The cultures were maintained under continuous light conditions of 12 h light: 12 h dark regime, with 2000 lux light intensity [4].

#### 3.1.2 Mass Culture in plastic jars

Filtered and autoclaved (for 15 mins in 121 °C and 15 lbs pressure) distilled water and sea water were used to prepare BBM and Conway media for mass culture sequentially. Growth media were prepared using required nutrient solutions; in case of marine stocks, salinity was maintained according to the salinity found in its sampling site. Pure stocks were then transferred to 20L plastic tanks for mass culture at 25 °C with a photoperiod of 12 h:12 h (light: dark), commencing with a 5L volume and eventually scaling up to around 16 L. Every two days, media was added to the culture tank until it reached the desired volume. Using a centralized air pump, individual culture tanks were continuously aerated. PVC pipe substrates were used for *Oscillatoria* sp. mass culture and samples from each tank were examined under a light microscope weekly to check the purity of the stock.

#### 3.1.3 Harvesting and dried biomass of Oscillatoria sp

After respective mass cultures, *Oscillatoria* were harvested by centrifugation at 5000 rpm for 5 minutes by using centrifugation machine (TL5R Free Standing low speed refrigerated centrifuge, Herexi). The wet microalgae biomass obtained from post-centrifugation was subsequently oven-dried overnight at 40 °C through a high-quality hot air oven (JSR Korea's Natural Convention Oven LNO-150) and the dried biomass was crushed into fine powders by using a mortar and pestle. The powdered microalgae were then stored in a standard freezer at 4 °C until further use.

### 3.2 Bacteria sampling site

Bacterial samples were collected from diseased marine fish namely Tuna (*Euthynnus affinis*), Rita (*Rita rita*), Gangetic Hairfin Anchovy (*Setipinna phasa*), Poa (*Otolithoides pama*), Tilapia (*Oreochromis mossambicus*) which were collected from Fishery Ghat, Chattogram, Bangladesh. Crustaceans such as Shrimp (*Penaeus monodon*) and Crab (*Scylla serrata*) were collected from the BFDC landing center and Crab farm, Cox's Bazar, Bangladesh respectively.

### 3.3 Collection and incubation of sample

Sterile cotton swab sticks were used to obtain samples from the diseased fish skin, gills, intestine and other affected areas. Sterile swabs were also used to swab the abdominal flap, mouthparts, claws, and carapace region of the crab, and the joints of the appendages, uropod, endoskeleton, and cephalothorax region of the shrimp which were subsequently inoculated into nutrient broth. The samples were then incubated overnight at 37 °C to allow the optimal growth and proliferation of the bacteria present in the samples.

### 3.4 Isolation and identification of bacteria by culture and staining

Bacterial culture from nutrient broth was streaked onto MacConkey agar, Mannitol Salt Agar (MSA), Thiosulfate citrate bile salts sucrose (TCBS), blood agar, Pseudomonas agar, Salmonella Shigella Agar (SS) agar and then incubated at 37 °C for 24 hours. The growth of *E. coli, Vibrio* sp., *Staphylococcus saprophyticus*, and *Aeromonas hydrophila* was indicated by bright-pink large colonies in MacConkey agar, green and yellow color colonies in TCBS agar, medium-sized yellow colonies in MSA, and round, smooth, and glistening colonies, looking like dewdrops with or without hemolysis in Blood agar, respectively. The bacteria were identified by further confirmation through biochemical tests.

### 3.5 Gram's staining

#### 3.5.1 Preparation of Smear

Gram's staining was carried out to identify the type of bacterium [5]. A sterile inoculating loop was used to transfer a drop of distilled water onto a glass slide, and it was combined with a loop full of bacterial culture that had been taken from agar plates. A room-temperature air drying period was subsequently given to the resultant smear. It was momentarily placed over a flame for the fixation of bacteria.

#### 3.5.2 Staining protocol

The principal stain crystal violet was added to the prepared slide and left on for one minute. To eliminate of any unbound crystal violet, the slide was washed with a gentle stream of water for up to two seconds. The mordant Gram's iodine was then used for 1 minute to attach the crystal violet to the bacterial cell wall. The slide was then rinsed for 2 seconds. Then acetone was applied for 15 seconds and washed with a gentle stream of water. Afterwards, the secondary stain safranin was applied to the slide for 1 minute and washed again with a gentle stream of water. Once the slide was air dried, Light microscopy was used to investigate it. Gram-negative bacteria displayed pink, but Gram-positive bacteria showed up as blue/purple.

### 3.6 Biochemical tests

Bacterial samples underwent biochemical assays using the VITEK 2 system at the Marine Biotechnology Laboratory, Universiti Malaysia Terengganu, Malaysia. *Aeromonas hydrophila* and *E. coli* were among the bacteria that the VITEK 2 system identified. Additional biochemical tests such as Catalase, Production of H_2_S, Triple Sugar Iron, and motility tests were conducted at the Disease and Microbiology laboratory, CVASU to confirm the presence of other bacteria.

### 3.7 Preparation of crude Oscillatoria spp. extract

2g dried cyanobacterial biomass was pulverized and sieved to obtain a fine powder, which was subsequently mixed with 20 ml 95 % pure ethanol (1g sample/10 ml ethanol) to yield the crude ethanolic extract [6]. It was soaked in the solvent in the sterile screw-capped bottles of 100 ml volume for a day (24 h) at room temperature. Then the obtained solution was fine filtered using Whatman No.1 filter paper under vacuum to remove cellular materials [7].

### 3.8 Extraction of Oscillatoria spp. phenolic compounds

Isolation of the potent extracts of phenolic compounds from *Oscillatoria* sp. was carried out in this study [8]. Filtered crude ethanolic extract solution was concentrated under reduced pressure through a rotary evaporator at their respective boiling points. The concentrated extract was then securely stored at 4 °C until subjected to the next step of extraction. For this, an acidic degradation method was employed [9] which involved addition of 2 ml of the concentrated extract to hydrochloric acid (2M, 200 ml) and heating the mixture in a water bath (90-100°C) with a stirrer for 30 minutes. After cooling the extract to room temperature, the solution was transferred to a separating funnel where ethyl acetate (100 ml) was added (this process was repeated twice). The two layers were then carefully separated, with the top layer comprising the ethyl portion rich in free phenolic acids. The extract was concentrated under pressure using a rotary evaporator, resulting in a precipitate which was stored at 4°C until it was ready for use in diagnosing phenols. Meanwhile, the aqueous layer was discarded.

### 3.9 Antibacterial activity of crude and phenolic extracts

The disk diffusion method was used to evaluate the antibacterial activity of the ethanolic raw extract and phenolic extract of *Oscillatoria* sp. [10], which involved Mueller-Hinton agar (MHA) plates. A turbidity of 0.5 McFarland standards was met by adjusting the bacterial suspension with 0.85 % physiological saline, which corresponded to approximately 1.5 × 10^8^ CFU/ml [11]. To assess the antimicrobial activity, six replicates of each bacterial species were inoculated onto MHA plates using sterile cotton swabs. To screen for inhibitory effects, sterile filter paper discs (6mm) were impregnated with 30 µl of the cyanobacterial extracts and then air-dried. Using sterilized forceps, the dried discs were placed on the Muller-Hinton agar plate. Colistin (10 µg/disc) was used as the standard antimicrobial disk for gram-negative bacteria such as *Aeromonas hydrophila, E. coli*, and *Vibrio* spp., *Salmonella, Shigella* sp., *Pseudomonas* spp., while Ceftriaxone (30 µg/disc) was used as the positive control for gram-positive bacteria including, *Streptococcus saprophyticus, Staphylococcus* spp. Sterile and clean Whatman filter paper was utilized as the negative control. The plates were then kept at 37 °C overnight while being inverted. The extracts created the distinct, circular inhibition zones surrounding the discs. Digital slide calipers (Robotics BD shipment) were used to measure the zone of inhibition from edge to edge.

### 3.10 Antimicrobial Activity Index (AI)

Comparison of the specific *Oscillatoria* sp. extract's zone of inhibition to that of a reference antibiotic, activity index (AI) was determined using the following equation [12,13]).

$$\begin{array}{c} Activity\;index=\frac{Diamter\;of\;the\;inhibition\;zone\;of\;extract}{Diamter\;of\;the\;inhibition\;zone\;of\;the\;reference\;antimicrobial} \end{array}$$

### 3.11 Minimal inhibitory concentration (MIC)

Minimum inhibitory concentration of the potent *Oscillatoria* sp. was evaluated by tube microdilution method [14]. 250 mg *Oscillatoria* sp. power was dissolved in 1 ml 100 % dimethyl sulfoxide (DMSO) and diluted with sterilized deionized water to 10 % DMSO. To achieve 1 % DMSO final concentrations, the stock solution was diluted with 10 % DMSO. After that, the samples were placed in a Sonicator machine for 10 minutes using pulsed, high frequency sound waves to agitate, and lyse the cells. The extract concentrations were then serially diluted to 300, 250, 200, 150, and 100 µg extracts/mL of 1 % DMSO. In a 96-well microtiter plate, each dilution was transferred using micropipette. Bacterial suspension was prepared and matched the standard of 0.5 McFarland. 150 µl bacterial suspensions were added in each well of a microtiter plate (96 well) from the column 1 to 12. Positive growth controls included both bacterial suspension and DMSO, whereas negative growth controls included DMSO with *Oscillatoria* extracts. Then the plates were incubated at 37 °C. Using a POLARstar Omega Plate from BMG LABTECH, Offenburg, Germany, the optical density of each well was measured spectrophotometrically at 630 nm. The lowest concentration of each test solution that prevented the development of any of the microorganisms in the wells was recorded as the MIC value following an overnight incubation. For each sample, the tests were carried out in triplicate, and the average concentration for each triplicate was determined.

### 3.12 Statistical analysis

All statistical analyses regarding the antimicrobial activity, activity index contents were performed by using the IBM SPSS (v. 26.0). Descriptive statistics were performed for each of the cyanobacteria for each of the parameters; then, a test for homogeneity of variance was performed. All of the collected data were analyzed by one-way analysis of variance (ANOVA) and significant differences amongst cyanobacterial species were analyzed using Tukey's multiple comparison tests at 95 % confidence interval level. Post-hoc test was utilized to discern differences between groups.

## Ethical Statement

No conflicts, informed consent, or human or animal rights are applicable to this study.

## CRediT Authorship Contribution Statement

**Jannatul Nayeem:** Methodology; Data collection; Data curation; Original draft. **Proma Dey, Sumit Kanti Dey:** Data curation. **Inkiad Ahmed Himel:** editing. **Helena Khatoon:** Conceptualization, project administration, supervision and review.

## Data Availability

A comprehensive dataset on antimicrobial activity of indigenous Oscillatoria spp. against common bacteria causing diseases in fish and shellfish (Original data) (Mendeley Data)
